# Breastfeeding and maternal health outcomes: a systematic review and meta-analysis

**DOI:** 10.1111/apa.13102

**Published:** 2015-11-04

**Authors:** Ranadip Chowdhury, Bireshwar Sinha, Mari Jeeva Sankar, Sunita Taneja, Nita Bhandari, Nigel Rollins, Rajiv Bahl, Jose Martines

**Affiliations:** 1 Centre for Health Research and Development, Society for Applied StudiesNew Delhi, India; 2 Newborn Health Knowledge Centre, ICMR Centre for Advanced Research in Newborn Health, Department of Paediatrics, All India Institute of Medical SciencesNew Delhi, India; 3 Department of Maternal, Newborn, Child and Adolescent Health, World Health OrganizationGeneva, Switzerland; 4 Centre for Intervention Science in Maternal and Child Health, Centre for International Health, University of BergenBergen, Norway

**Keywords:** Breastfeeding, Long and Short Term, Maternal health, Meta-analysis

## Abstract

**Aim:**

To evaluate the effect of breastfeeding on long-term (breast carcinoma, ovarian carcinoma, osteoporosis and type 2 diabetes mellitus) and short-term (lactational amenorrhoea, postpartum depression, postpartum weight change) maternal health outcomes.

**Methods:**

A systematic literature search was conducted in PubMed, Cochrane Library and CABI databases. Outcome estimates of odds ratios or relative risks or standardised mean differences were pooled. In cases of heterogeneity, subgroup analysis and meta-regression were explored.

**Results:**

Breastfeeding >12 months was associated with reduced risk of breast and ovarian carcinoma by 26% and 37%, respectively. No conclusive evidence of an association between breastfeeding and bone mineral density was found. Breastfeeding was associated with 32% lower risk of type 2 diabetes. Exclusive breastfeeding and predominant breastfeeding were associated with longer duration of amenorrhoea. Shorter duration of breastfeeding was associated with higher risk of postpartum depression. Evidence suggesting an association of breastfeeding with postpartum weight change was lacking.

**Conclusion:**

This review supports the hypothesis that breastfeeding is protective against breast and ovarian carcinoma, and exclusive breastfeeding and predominant breastfeeding increase the duration of lactational amenorrhoea. There is evidence that breastfeeding reduces the risk of type 2 diabetes. However, an association between breastfeeding and bone mineral density or maternal depression or postpartum weight change was not evident.

## Introduction

Breast milk is the natural first food for newborns. It provides all the energy and nutrients that an infant needs for the first six months of life, up to half or more during the second half of infancy and up to one-third during the second year of life (1,2). For mothers, breastfeeding has been reported to confer lower risk of breast and ovarian carcinoma (3,4), greater postpartum weight loss (5) and decreased blood pressure (6) compared with no breastfeeding. The World Health Organization (WHO) recommends exclusive breastfeeding in the first six months and continuation of breastfeeding for 2 years and beyond (1).

The association between breastfeeding and breast carcinoma in mothers has received increased scrutiny in recent years. A number of studies have suggested that breastfeeding, particularly for an extended period of time, may be associated with a decreased risk of breast carcinoma, even after adjustment for potential confounders (7). It is difficult, however, to estimate the magnitude of association between breastfeeding duration and breast carcinoma if any, because of the different methodologies used in breastfeeding histories. Parity is also a protective factor against breast carcinoma (8), and there may be an interaction between parity and breastfeeding duration interplay in protecting women from breast carcinoma.

**Key Notes**

- Longer duration of breastfeeding protects against breast and ovarian carcinoma.
- Exclusive breastfeeding and predominant breastfeeding increase the duration of lactational amenorrhoea.
- Evidence on the association between breastfeeding and maternal bone mineral density, maternal depression or postpartum weight change was lacking.

Ovarian cancer is one of the most common cancers in female (9,10). Reproductive factors have been identified as markers of risk for ovarian cancer. These reproductive factors mainly include total number of pregnancies, parity, age at menarche and menopause, as well as breastfeeding (11). Evidence from previous analyses indicates an inverse association between breastfeeding and the risk of ovarian carcinoma (4,12).

Calcium metabolism and bone metabolism are substantially altered with increased calcium demands during pregnancy and lactation. Bone densities can decrease by between 3 and 10 per cent in the span of a few months in a healthy mother (13). Confounders commonly considered in the studies of the relationship between fracture risk and breastfeeding are age, hormone replacement therapy, parity and BMI (4).

Available literature suggests that breastfeeding reduces the risk of maternal type 2 diabetes in some cohort studies, but the evidence from published studies has differed with regard to the strength of the association (14,15).

The literature suggests that exclusive breastfeeding protects against pregnancy (16,17). Some studies, however, show that exclusive breastfeeding is not always associated with inhibition of ovulation (18,19).

The incidence of postpartum depression (PPD) is high (10–15%) (20), and depression during pregnancy usually continues into the postpartum period (21). Postpartum depression has an immediate impact on mothers. It carries long-term risks for their mental health (22) and may also have significant negative effects on the cognitive, social and physical development of their children (23). The evidence for an association between breastfeeding and PPD is, however, unclear (23,24).

Postpartum weight retention is a predictor for future overweight and obesity (25) and is associated with obesity-related illnesses, such as type 2 diabetes mellitus and cardiovascular disease (26). Breastfeeding may promote weight loss due to lactation (27), but there is a lack of strong evidence to support this hypothesis (28).

We conducted this review to summarise the literature and explore the relationship of breastfeeding and its duration with long-term (breast carcinoma, ovarian carcinoma, osteoporosis and type 2 diabetes mellitus) and short-term (lactational amenorrhoea, postpartum depression, postpartum weight change) maternal health outcomes. Outcomes for review were selected during an expert meeting at the World Health Organization (October 2014) that was reviewing the impact of breastfeeding on maternal and child health.

## Methods

A search strategy (Box 1) was developed and reviewed by all authors. Medical Subject Heading (29) terms and keywords were used in various combinations. We searched published literature from PubMed, Cochrane Library and CABI databases to identify studies examining the effect of type and duration of breastfeeding on maternal health outcomes. We conducted the search in February 2015. No language or date restrictions were employed in the electronic search.

**Box 1. Search strategy for breastfeeding & maternal health**

1. Breastfeeding OR Breast Feeding OR Lactation OR Human Milk OR Breast Milk
2. Women OR Maternal OR Postpartum OR puerperal OR postnatal OR Birth OR gestation
3. Diabetes OR (Breast AND (Carcinoma OR carcinoma OR tumor OR malignancy)) OR (Ovarian OR Ovary AND (Carcinoma OR carcinoma OR tumor OR malignancy)) OR (depression OR Blues OR psychosis) OR (Amenorrhea OR Contraception) OR (Osteoporosis OR Bone mineral density) OR Weight OR BMI OR body mass index
4. (Addresses[ptyp] OR Autobiography[ptyp] OR Bibliography[ptyp] OR Biography[ptyp] OR pubmed books[filter] OR Case Reports[ptyp] OR Congresses[ptyp] OR Consensus Development Conference[ptyp] OR Directory[ptyp] OR Duplicate Publication[ptyp] OR Editorial[ptyp] OR Festschrift[ptyp] OR Guideline[ptyp] OR *In Vitro*[ptyp] OR Interview[ptyp] OR Lectures[ptyp] OR Legal Cases[ptyp] OR News[ptyp] OR Newspaper Article[ptyp] OR Personal Narratives[ptyp] OR Portraits[ptyp] OR Retracted Publication[ptyp] OR Twin Study[ptyp] OR Video-Audio Media[ptyp])
5. #1 AND #2 AND #3
6. #5 NOT #4

Two review authors (RC and BS) screened the titles and abstracts independently to identify potentially relevant citations. These review authors retrieved the full texts of all potentially relevant articles and independently assessed the eligibility of the studies using predefined inclusion criteria. We extracted data from all articles found to be relevant by both authors. Any disagreements or discrepancies between reviewers were resolved by discussion and if necessary by consulting a third author (JSM). In addition to the electronic search, we searched reference lists of the articles identified. We used Web-based citation index for citing manuscripts of these identified articles.

We identified four recent systematic reviews addressing the following outcomes: ovarian carcinoma (30), type 2 diabetes mellitus (31), postpartum depression (32) and postpartum weight change (33). We planned to update these reviews and provide new quantitative estimates of breastfeeding on these health outcomes. For other maternal health outcomes, that is breast carcinoma, osteoporosis and lactational amenorrhoea, we planned for new reviews.

### Inclusion criteria

We selected all observational studies (prospective/retrospective cohort and case–control), randomised controlled trials (RCTs), including cluster randomised trials, and quasi-experimental trials which examined the impact of duration and type of breastfeeding on maternal health outcomes. For articles not written in English, we attempted to get an English abstract. If it was not available, the article was excluded.

### Abstraction, summary measure, breastfeeding categories and analysis

We abstracted data using a modified Cochrane data abstraction form. If a study provided separate estimates for hospital- and community-based populations, then the outcome estimates were pooled separately. We used odds ratios (ORs), both adjusted and unadjusted, as our outcome estimate for breast and ovarian carcinoma. Relative risk (RR) was used as the outcome estimate for lactational amenorrhoea. To examine the effect on breast and ovarian carcinoma, breastfeeding was categorised into ever breastfed vs. never breastfed and also by breastfeeding duration, that is breastfed less than six months vs. not breastfed; breastfed 6 to 12 months vs. not breastfed; and breastfed >12 months vs. not breastfed. For lactational amenorrhoea, we used exclusive, predominant, partial, any and no breastfeeding as the categories (Table A1). Standardised mean differences in bone mineral density between highest and lowest breastfeeding duration categories were used for osteoporosis outcome. A narrative approach was used to summarise the studies for postpartum weight change as the studies were very heterogeneous.

We performed meta-analysis with Stata 11.2 software (StataCorp, College Station, TX, USA). We calculated the pooled estimates of the outcome measures from the odds ratios (ORs), relative risks (RRs), standardised mean differences (SMDs) and 95% confidence intervals (CIs) of the individual studies by inverse variance or DerSimonian and Laird method in Stata (34). High heterogeneity was defined by either a low p-value (<0.10) or *I*^2^ value greater than 60%. In cases of high heterogeneity, the random-effects model was used and causes were explored by conducting subgroup analysis and meta-regression. Subgroup analyses were carried out based on breastfeeding categories (ever vs never, less than six months vs never, 6–12 months vs never, >12 months vs never). Among the ever vs never breastfeeding category, subgroup analyses were carried out based on sample size (<500, 500–1499, ≥1500), individual study setting (i.e. high-income country (HIC) or low- and middle-income country (LMIC) (35)), study design (cohort, case–control), mean age of diagnosis (≤49 years, >49 years), adjustment for parity (fine adjustment, i.e. adjustment according to each parity number measured as 0, 1, 2, 3, 4+; crude adjustment, i.e. groupwise adjustment measured as 0, 1–3, 4+ children; and no adjustment), control for confounding (thorough, i.e. controlled for all potential socio-demographic and reproductive factors such as age, income, ethnicity, parity, contraceptive use, family history of carcinoma, menopausal status and smoking; partial, i.e. only partially controlled for potential socio-demographic and reproductive factors; and none) and quality of study (adequate, i.e. study had none or one among selection bias, measurement bias, attrition (20%) and confounding bias; inadequate) (36). We also evaluated the presence of publication bias in the extracted data for the primary outcome using Begg's test or Egger's test or funnel plots (37).

## Results

**Figure 1:**
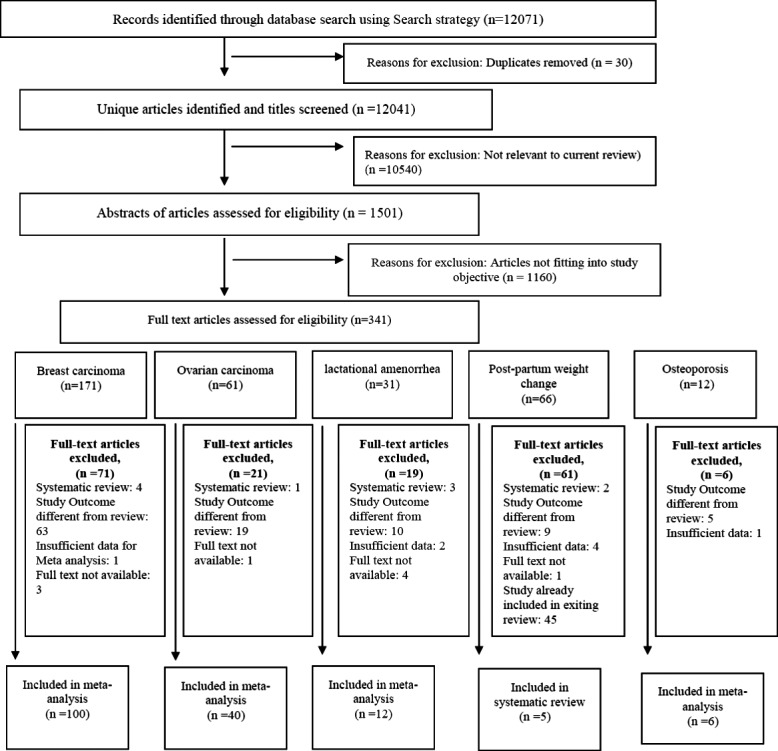
: Prisma Flow chart.

We screened the 12 071 titles identified. Of these, after reviewing abstracts of 1501 articles, we selected 341 for full-text review. We identified 163 articles for inclusion in our final database (Fig.1). Among these, 100 studies examined the impact of breastfeeding on breast carcinoma, 40 studies on ovarian carcinoma, 12 studies on lactational amenorrhoea, five studies on postpartum weight change and six studies on osteoporosis. We did fresh meta-analysis for breast carcinoma, ovarian carcinoma, osteoporosis and lactational amenorrhoea and updated the review on postpartum weight change. No new studies subsequent to the existing reviews on type 2 diabetes mellitus and postpartum depression (31,32) were found to be eligible for inclusion.

### Effects of breastfeeding on long-term maternal health outcomes

#### Breast carcinoma

**Figure 2:**
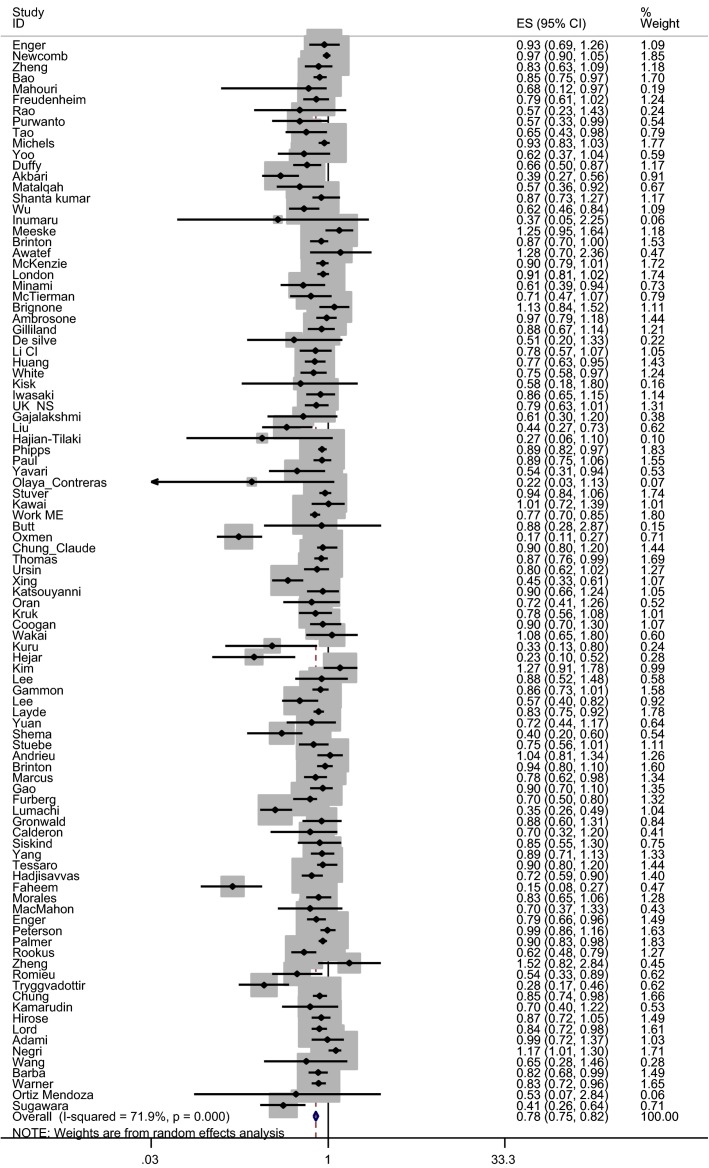
Effect of ever breastfeeding vs. no breastfeeding on risk of breast carcinoma.

We identified 98 estimates (38–135) of the association between ever breastfeeding and breast carcinoma risk (Tables 1 and A2). Ever breastfeeding was associated with 22% (OR 0.78, 95% CI 0.74–0.82) (Fig.2) reduction of breast carcinoma risk compared with never breastfeeding. Compared with no breastfeeding, breastfeeding for less than six months (39 estimates) and breastfeeding for 6–12 months (36 estimates) were associated with 7% (OR 0.93, 95% CI 0.88–0.99) and 9% (OR 0.91, 95% CI 0.87–0.96) risk reduction of breast carcinoma, respectively. We found that mothers who breastfed for >12 months compared with those who did not breastfeed had a 26% lower risk of developing breast carcinoma (50 studies; OR 0.74, 95% CI 0.69–0.79), and when restricted to high-quality studies, only (41 studies) breastfeeding >12 months was associated with 23% lower risk of developing breast carcinoma (OR 0.77, 95% CI 0.72–0.83) (not shown in Table 1). There was, however, an indication of publication bias. Asymmetry was observed in funnel plot when inspected visually. Both Egger's test (p bias <0.001) and Begg's test (p bias <0.001) showed statistically significant findings.

**Table 1:** Risk of breast carcinoma by breastfeeding duration and by subgroup

	Number of estimates	Pooled odds ratio and 95% confidence interval	p-value	*I*^2^ (%)	
Breastfeeding category					
Ever vs. Never	98	0.78 (0.74; 0.82)	<0.001	71.9	
<6 months vs. Never	39	0.93 (0.88; 0.99)	0.05	59.1	
6–12 months vs. Never	36	0.91 (0.87; 0.96)	<0.001	22.5	
>12 months vs. Never	50	0.74 (0.69; 0.79)	<0.001	62.2	

Subgroup analysis (Ever vs. Never)	Number of estimates	Pooled odds ratio and 95% confidence interval	p-value	*I*^2^ (%)	Meta-regression p-value
Study size					
<500 participants	15	0.50 (0.37; 0.66)	<0.001	59	0.009
500–1499 participants	31	0.74 (0.66; 0.83)	<0.001	66.7	
≥1500 participants	52	0.83 (0.80; 0.88)	<0.001	71.7	
Setting					
High income	72	0.81 (0.77; 0.85)	<0.001	72.5	0.206
Lower mid-income	26	0.66 (0.56; 0.77)	<0.001	68.3	
Study design					
Cohort	12	0.85 (0.83; 0.87)	<0.001	53.5	0.705
Case–control	86	0.77 (0.72; 0.81)	<0.001	73.3	
Mean age					
≤49	28	0.78 (0.71; 0.87)	<0.001	73.5	0.369
>49	28	0.68 (0.60; 0.78)	<0.001	84.8	
Adjusted for parity					
Fine adjustment	19	0.92 (0.88; 0.96)	<0.001	54.8	0.037
Crude adjustment	19	0.86 (0.81; 0.90)	<0.001	23.2	
No adjustment	60	0.73 (0.68; 0.79)	<0.001	77.4	
Control for confounding					
Thorough	40	0.82 (0.77; 0.87)	<0.001	68	0.479
Partial	25	0.77 (0.69; 0.87)	<0.001	71.2	
None	33	0.74 (0.68; 0.81)	<0.001	76.1	
Quality of study					
Adequate	66	0.81 (0.78; 0.85)	<0.001	62.6	0.750
Inadequate	32	0.70 (0.61; 0.80)	<0.001	81.6	

Subgroup analysis of the effects of ever breastfeeding on risk of breast carcinoma among studies conducted in high-income countries, with large sample sizes (>1500), of cohort design, with thorough control of confounding factors and adequate quality showed a smaller breast carcinoma risk reduction. Studies where fine adjustment for parity was made showed a smaller effect of breastfeeding on breast carcinoma risk reduction (OR 0.92, 95% CI 0.88–0.96) compared with studies where crude adjustment or no adjustment was made. A restricted analysis including parous women in the fine adjustment subgroup showed a risk reduction of 7% for breast carcinoma (OR 0.93, 95% CI 0.89–0.97; 14 estimates) (not shown in Table 1).

#### Ovarian carcinoma

**Figure 3:**
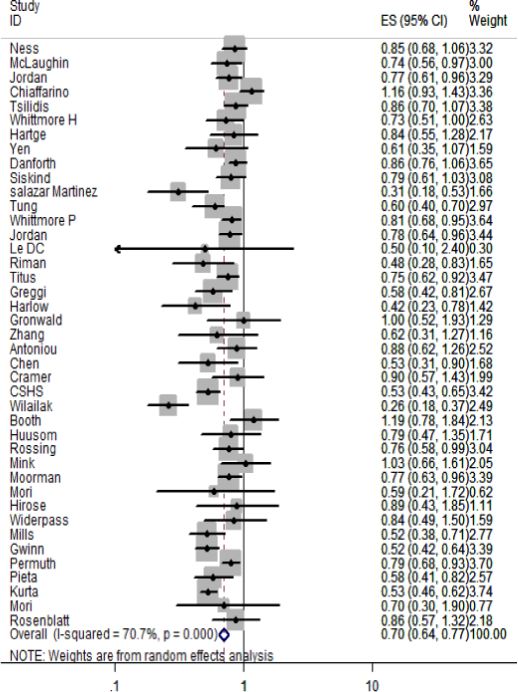
Effect on ever vs. never breastfeeding on risk of ovarian carcinoma.

Pooled results from 41 estimates (65,69,136–173) showed that mothers who ever breastfed their children had a 30% reduction in the risk of ovarian carcinoma, when compared with those who never breastfed (OR 0.70, 95% CI 0.64–0.77) (Tables 2 and A3; Fig.3). The risk of ovarian carcinoma was 17% lower among women who had breastfed for less than six months when compared with those who did not breastfeed (OR 0.83, 95% CI 0.78–0.89). The risk of ovarian carcinoma among mothers who breastfed for 6–12 months was 28% lower (OR 0.72, 95% CI 0.66–0.78; 19 estimates) when compared with women who had not breastfed. The highest risk reduction was observed among women who breastfed for more than 12 months, in whom the risk of ovarian carcinoma was 37% lower than among women who had not breastfed (OR 0.63; 95% CI 0.56–0.71; 29 estimates). The effect size was slightly less (OR 0.65, 95% CI 0.57–0.73), when the analyses were restricted to high-quality studies (29 estimates). There was no evidence of publication bias in Egger's test or Begg's test (p bias >0.1) in either of the analyses.

**Table 2:** Risk of ovarian carcinoma by breastfeeding duration and by subgroup

	Number of estimates	Pooled odds ratio and 95% confidence interval	p-value	*I*^2^ (%)	
Breastfeeding category					
Ever vs. Never	41	0.70 (0.64; 0.77)	<0.001	70.0	
<6 months vs. Never	20	0.83 (0.78; 0.89)	<0.001	3.0	
6–12 months vs. Never	19	0.72 (0.66; 0.78)	<0.001	22.0	
>12 months vs. Never	29	0.63 (0.56; 0.71)	<0.001	51.8	

Subgroup analysis (Ever vs. Never)	Number of estimates	Pooled odds ratio and 95% confidence interval	p-value	*I*^2^ (%)	Meta-regression p-value
Study size					
<500 participants	7	0.67 (0.53; 0.84)	0.001	2.8	0.241
500–1499 participants	12	0.59 (0.47; 0.74)	<0.001	74.9	
≥1500 participants	22	0.76 (0.69; 0.84)	<0.001	70.9	
Setting					
High income	35	0.74 (0.68; 0.80)	<0.001	64.0	0.007
Lower mid-income	6	0.48 (0.29; 0.77)	<0.001	77.1	
Study design					
Cohort	5	0.87 (0.78; 0.98)	0.02	0.0	0.136
Case–control	36	0.68 (0.61; 0.75)	<0.001	71.6	
Mean age					
≤49	10	0.70 (0.64; 0.77)	<0.001	70	0.744
>49	24	0.71 (0.63; 0.80)	<0.001	77	
Adjusted for parity					
Fine adjustment	16	0.80 (0.75; 0.86)	<0.001	56	0.231
Crude adjustment	4	0.69 (0.59; 0.81)	<0.001	45	
No adjustment	21	0.67 (0.58; 0.77)	<0.001	72	
Control for confounding					
Thorough	14	0.76 (0.67; 0.85)	<0.001	64.9	0.419
Partial	15	0.66 (0.55; 0.81)	<0.001	73	
None	11	0.72 (0.65; 0.78)	<0.001	50.5	
Quality of study					
Adequate	27	0.72 (0.65; 0.80)	<0.001	71	0.505
Inadequate	14	0.63 (0.58; 0.68)	<0.001	54.7	

In subgroup analysis, studies with sample sizes of more than 1500 showed a significant protection of 24% from ovarian carcinoma (OR 0.76, 95% CI 0.69–0.84). This effect size was reduced compared to studies with smaller samples (OR 0.67, 95% CI 0.53–0.84). Studies in HICs also showed a significant but reduced effect (OR 0.74, 95% CI 0.68–0.80) compared with studies in LMICs (OR 0.48 95% CI 0.29–0.77). Lower quality studies showed a higher risk reduction for ovarian carcinoma (OR 0.63, 95% CI 0.58–0.68) than higher quality studies (OR 0.72, 95% CI 0.65–0.80). Studies where fine adjustment for parity was made showed a modest but still significant (OR 0.80, 95% CI 0.75–0.86) reduction in risk of ovarian carcinoma compared with studies where no or crude adjustment for parity was made. In an analysis restricted to parous women in the fine adjustment subgroup, the effect was further attenuated (OR 0.82, 95% CI 0.75–0.89) (not shown in Table 2).

#### Osteoporosis

A total of six studies (174–179) were identified (Table 3). Two studies were from LMICs (174,178) and four studies from HICs (175–177,179). Bone mineral density (BMD) was generally measured at two sites, that is femoral neck and distal radius. For femoral neck, four studies (175,177–179) were identified with small sample size (total 489 women). The pooled effect suggests that breastfeeding had a nonsignificant effect on femoral neck bone mass. With respect to distal radius, four studies (174–177) were identified and the results were heterogeneous. The largest (n = 963) study (176) did not observe any association, whereas Chowdhury et al. (174) (n = 400) reported a negative effect of breastfeeding on bone mineral density. Overall, there was no clear evidence of an effect of breastfeeding on osteoporosis.

**Table 3:** Association between breastfeeding and bone mineral density

First author name (year)	Mean ± SD: BMD highest BF group (g/cm^2^)	Mean ± SD: BMD lowest BF group (g/cm^2^)	Pooled SMD (95% CI) of BMD
Distal Radius			
Chowdhury (2002) (174)	0.49 ± 0.11	0.61 ± 0.08	Fixed effect −0.132 (−0.26 to −0.003)
Hawker (2002) (176)	0.477 ± 0.05	0.474 ± 0.03	
Henderson (2000) (177)	0.564 ± 0.06	0.601 ± 0.05	Random effect −0.490 (−1.357 to 0.376)
Drinkwater (1991) (175)	0.541 ± 0.07	0.545 ± 0.05	
Femoral Neck			
Henderson (2000) (177)	0.835 ± 0.11	0.847 ± 0.12	Fixed effect −0.142 (−0.426 to 0.142)
Lenora (2009) (178)	0.603 ± 0.13	0.613 ± 0.12	
Wiklund (2012) (179)	0.96 ± 0.11	0.97 ± 0.11	
Drinkwater (1991) (175)	0.95 ± 0.05	1.01 ± 0.10	

BMD, bone mineral density; SD, standard deviation; SMD, standardised mean difference.

#### Diabetes

A recent systematic review by Aune reported a reduced risk of type 2 diabetes (RR 0.68 95% CI: 0.57–0.82) with longer duration of lifetime breastfeeding compared with shorter durations. A one-year increase in the total lifetime duration of breastfeeding was associated with 9% protection (RR 0.91, 95% CI: 0.86–0.96) against the presence of type 2 diabetes in the mothers. No new studies were found subsequent to the systemic review by Aune et al. in 2013.

### Effects of breastfeeding on short-term maternal health outcomes

#### Lactational amenorrhoea

We identified 12 studies (173,180–190) that examined the association between breastfeeding and lactational amenorrhoea (Table 4). Four studies (180,182,188,185) did not provide either RR or OR. They reported that exclusive compared to mixed feeding, or longer duration of any breastfeeding, was associated with an increased mean or median duration of lactational amenorrhoea. The remaining studies provided data from which the following estimates were derived: the probability of continued lactational amenorrhoea at six months postpartum was 23% higher (RR 1.23, 95% CI 1.07–1.41; three studies) for exclusive or predominant breastfeeding compared to no breastfeeding, and 21% higher (RR 1.21, 95% CI 1.01–1.25; five studies) (Table 4) when compared to partial breastfeeding. We found no evidence of publication bias.

**Table 4:** Effect of breastfeeding on probability of lactational amenorrhoea

Breastfeeding category	No. of Estimates	Ref. no	Probability of lactational amenorrhoea RR (95% CI)	p-value (test of heterogeneity)
Exclusive or Predominant BF vs. No BF	03	(176,180,185)	1.23 (1.07–1.41)	0.34
Exclusive or Predominant BF vs. Partial BF	05	(174,178,179,182,184)	1.21 (1.01–1.25)	0.08
Any BF (exclusive or predominant or partial BF) vs. No BF	04	(181,185,186,190)	1.14 (0.92–1.40)	0.01

#### Postpartum depression

A recent systematic review conducted by Dias et al. reported that pregnancy depression predicts a shorter breastfeeding duration, but evidence is unclear on whether breastfeeding mediates the association between pregnancy and postpartum depression. No new studies were found subsequent to the systemic review conducted by Dias and Figueiredo in (32).

#### Postpartum weight change

We updated the systematic review by Neville et al. (33) by including 5 additional studies (Table 5) (191–195). In the review by Neville et al., the majority of identified studies reported little or no association between breastfeeding and weight change. Of those five studies, three studies were performed in low- and middle-income countries, one was performed in high-income country, and one was multicentre study (Brazil, Ghana, India, Norway, Oman, USA). In studies performed in low- and middle-income countries, we have not found any potential differential effect for breastfeeding and postpartum weight loss response as a function of countries being low to middle and high income. Two of the five additionally identified studies (194,195) reported a significant reduction in postpartum weight with breastfeeding. Sarkar and Taylor (191) in a cross-sectional study in Bangladesh revealed that body weight of mothers was negatively correlated with 1–12 and 13–24 months of lactation after controlling for height, education and food consumption. Stuebe et al. (192) showed that women who exclusively breastfed for greater than six months had the lowest BMI at 3 years postpartum as well as the lowest postpartum weight retention at 3 years compared with women who never exclusively breastfed. A multicentre study showed that lactation intensity and duration explained little variation in weight change patterns (193–195). Overall, the role of breastfeeding on postpartum weight change remains unclear.

**Table 5:** Overview of studies which examined the association between breastfeeding and postpartum weight change

S. No.	Author Name (year)	Location	Age		Association between Breastfeeding and Weight Change			Covariates included in analyses
1	Monteiro et al. 2013 ((194))	Brazil	<24 years – 48.2% >24 years – 51.8%		For women within 2-year postpartum period, each breastfeeding score point increases an average postpartum loss of 70 g (p = 0.002)			Sanitary condition of household
								Social programs of income transfer
								Type of delivery
								Prepregnancy weight
2	Onyango et al. 2011 ((193))	Brazil, Ghana, India, Norway, Oman, USA	Brazil	28.3 (6.3)	Lactation intensity and duration explained little of the variation in weight change patterns			Maternal age
			Ghana	30.8 (3.9)				BMI at 14 days
			India	29.0 (3.5)				Parity
			Norway	30.8 (4.3)				Delivery mode
			Oman	27.7 (5.0)				Infant birthweight
			USA	31.5 (4.5)				Sex
3	Samano et al. 2013 ((195))	Mexico	18.8 years		Among both adult and adolescent mothers, those who practised EBF lost more weight than those who did not practise EBF (−2.9 kg, interquartile range, −5.7 to 0.8 kg, vs −1.8 kg, interquartile range −2.8 to 2.2 kg)			Pregestational weight
								Marital status
								Education
								Delivery mode
					Duration of Lactation	Mean Body Weight (kg)	Mean BMI (kg/m^2^)	
4	Sarkar et al. 2005 ((191))	Bangladesh	18–40 years		Nonlactating	44.3	19.4	Age Height Education
					<12	42.48	18.86	
					12–24	42.96	18.85	
					25–36	43.32	19.11	
					37–48	43.47	19.29	
					49–60	44.11	19.36	
5	Stube et al. 2010 ((192))	East Massachusetts, USA	Ghana: 30.8 (3.9)		Months of Lactation	BMI kg/m^2^ (95% CI)		Prepregnancy BMI
					0	26.1 (25.4–26.9)		Parity
					0–3	25.3 (24.6–26.0)		Family history of diabetes
					3–6	25.5 (24.9–26.2)		
					6–12	25.8 (25.2–26.3)		
					>12	25.4 (24.8–26.0)		
					Months of Lactation	Wt. retention kg mean (95% CI)		
					0	2.5 (0.6–4.3)		
					0–3	0.2 (−1.6–2.0)		
					3–6	0.9 (−0.8–2.6)		
					6–12	1.3 (−0.1–2.8)		
					>12	0.4 (−1.2–1.9)		

## Discussion

The aim of this review was to systematically examine the effect of breastfeeding on important maternal health outcomes.

The risk of developing breast carcinoma was reduced by 26% among women who cumulatively breastfed for more than 12 months, compared with women who did not breast feed.

Previous reviews suggested that breastfeeding was not strongly related to risk of breast carcinoma (196,197) or found a small but statistically significant protective association (198–200). Our meta-analysis findings are comparable with but suggest a higher level of protection than that found by the Collaborative Group on Hormonal Factors in Breast Carcinoma (201). In this pooled analysis of approximately 50 000 carcinoma cases from 47 studies in 30 countries around the world and after adjustment for confounders including parity and exclusion of nulliparous women, the authors estimated that the risk of invasive breast carcinoma decreased by 4.3% for every 12 months of breastfeeding (201). However, one of the challenges of comparing studies on cumulative breastfeeding duration and determining the effect on breast carcinoma risk is the lack of a standard protocol for grouping the lifetime number of months of breastfeeding for analysis and the adjustment of parity. Lifetime duration of breastfeeding is related to the number of children breastfed, that is parity and the duration of breastfeeding for each child. Our results showed that when controlled for parity, breastfeeding independently contributed to a modest but significant risk reduction for breast carcinoma. The risk reduction for breast carcinoma was 8% among ever breastfed mothers when finely adjusted for parity, while it was 22% when all studies were pooled together. Even when our analysis was restricted to only parous women, finely adjusted for parity, ever breastfeeding was associated with a 7% reduction in risk of breast carcinoma compared with never breastfeeding. Longer duration of breastfeeding (>12 months) was associated with more protection of breast carcinoma than shorter duration of breastfeeding (breastfeeding <6 and 6–12 months) when compared to never breastfeeding. Even when our analysis was restricted to studies with adequate quality, breastfeeding >12 months showed more protection against breast carcinoma. Possible biological mechanisms include that protection may occur through parity-specific changes in levels of circulating hormones such as estradiol, prolactin and growth hormone, as each of these has been associated with breast cancer risk (202), or that the parous mammary gland may contain epithelial cells with a more differentiated and less proliferative character which are less susceptible to transformation (203).

Breastfeeding by women for more than 12 months was also associated with a 35% reduction in ovarian cancer, compared with women who had not breastfed. The protective effect was less in women who had only ever breastfed (for any duration) ranging from 30% in an unadjusted analysis to 18% when the analysis was restricted to ever breastfeeding parous women (finely adjusted for parity). A number of physiological mechanisms may account for the protective effect of breastfeeding against ovarian cancer through modulating ovarian cycle length (204), and therefore, parity is an important confounder. Longer duration of breastfeeding suppresses ovulation longer and causes suppression of gonadotropins, resulting in depressed production of plasma estradiol, considered to be a potential causal mechanism of ovarian cancer when present at high levels (205). However, breastfeeding must also have an independent effect to explain the estimated reduction in ovarian cancer when parity is adjusted for.

There did not appear to be a significant effect of breastfeeding on the risk of osteoporosis. Calcium metabolism and bone metabolism are substantially altered during pregnancy and lactation, and high calcium demand during lactation makes women more prone to bone resorption and subsequent osteoporosis. There was no evidence of such risk, and it has been suggested that during lactation, oestrogen imposes minor inhibitory effect on periosteal bone formation and permits periosteal expansion which increases bone size after weaning (206).

Available review suggests that longer duration of breastfeeding reduces risk of development of type 2 diabetes mellitus by 32%, and in linear dose–response analyses, there was a 9% reduction in relative risk for each 12-month increase in lifetime duration of breastfeeding. Our review shows that exclusive or predominant breastfeeding during the first six months postpartum was associated with longer periods of amenorrhoea. Less intensive breastfeeding, captured under ‘any or partial breastfeeding’, offers less clear benefit. This finding is biologically plausible. Breastfeeding suppresses the resumption of ovarian activity after childbirth and is thus associated with a period of infertility. Exclusive breastfeeding and predominant breastfeeding are associated with a higher frequency of suckling than other patterns of breastfeeding. Frequent suckling inhibits gonadotropin-releasing hormone and decreases the release of luteinising hormone and follicle-stimulating hormone (207), thus preventing early return of menses.

The association between breastfeeding and postpartum weight change remains uncertain. Factors such as age, gestational weight gain and prepregnancy weight confound such analyses (208,209). As prepregnancy weight and gestational weight gain were found to be strong determinant factors of postpartum weight change, future research should include the preconception period with continued monitoring into the postpartum period to capture the true trajectory of weight change. Even though BF may not lead to postpartum weight loss under ‘natural’ conditions, it remains unknown whether women who wish to lose weight intentionally in the postpartum period are more likely to be successful at doing so if they are vs. if they are not breastfeeding.

Although our original review plans included exploring the associations between breastfeeding and the risk of maternal postpartum depression and type 2 diabetes, we were unable to identify new studies following the reviews published in 2015 (31) and 2013 (32). The evidence suggests that the relationship between breastfeeding and postpartum depression is lacking.

The range of the maternal outcomes examined and the various categories of breastfeeding exposures that we considered are important strengths of this review. Despite the expanded scope of review, other important maternal health outcomes such as maternal hypertension and cardiovascular disease were not addressed and should be considered in future research and reviews. Also important was the attempt to look for dose–response relationships and the evaluation of heterogeneity and publication biases. However, some limitations should be acknowledged. We have pooled data from many observational studies that are prone to be affected by biases such as in recall or due to selection. Some studies did not control for or collect information on potential confounders that could have affected the association between breastfeeding and the outcome of interest. For postpartum weight change, we were constrained to take a narrative approach to present the outcomes because of the heterogeneous nature of the studies. In cases of significant heterogeneity in study results, we have performed post hoc subgroup analysis and meta-regression and have used the random-effects model. But in some cases even within subgroups, there was significant heterogeneity which suggests some other unidentified factors causing such heterogeneity. Although the meta-regression seemed to explain around 80% of the heterogeneity for breast and ovarian carcinoma, we need to acknowledge the limitation of post hoc subgroup analysis.

## Conclusion

Our meta-analysis shows that women who had ever breastfed and who breastfed for longer duration have a lower risk of breast and ovarian carcinoma and also type 2 diabetes mellitus. Exclusive or predominant breastfeeding during the first six months postpartum prolongs lactational amenorrhoea. We found no evidence of a clear association between breastfeeding and bone mineral density, maternal depression or postpartum weight change.

## Appendix

**Table A1:** Breastfeeding exposures (WHO definitions) (210)

Exposure Category	Permitted to Receive
Exclusive breastfeeding	• Breast milk from mother or wet nurse or expressed breast milk
	• No other liquids or solids except vitamin drops or syrups, mineral supplements, or prescribed medicines
Predominant breastfeeding	• Breast milk from mother or wet nurse or expressed breast milk
	• Water and water-based drinks
	• No food-based fluid with the exception of fruit juice and sugar water
	• Vitamin drops or syrups, mineral supplements, or prescribed medicines
Partial breastfeeding	• Breast milk from mother or wet nurse or expressed breast milk
	• Any other liquids or nonliquids, including both milk and nonmilk products
Any breastfeeding	• Breast milk from mother or wet nurse or expressed breast milk
	• Includes children exclusively, predominantly, fully and partially breastfed
No breastfeeding	• Formula and/or animal's milk
	• No breast milk

**Table A2:** Summary of studies included in breast carcinoma

Estimates	Studies	Ref. No.	Design		Country		Quality	
Ever vs. Never								
98	98	(38–135)	Cohort	12	HIC	72	AQ	66
			Case–control	86	LMIC	26	IQ	32
<6 months vs. Never								
39	39	(39,40,42,48,51,53,56,57,59,60,62,66,69,71,74,78,80,83,85,88,92,93,95,96,98,99,105,107,110,112,114–116,119–121,135,211,212)	Cohort	7	HIC	33	AQ	32
			Case–control	32	LMIC	6	IQ	7
6–12 months vs. Never								
36	36	(39,42,48,51,56,57,59,60,62,63,66,69,71,74,78,80,83,85,88,93,95,96,98,99,105,107,110,112,114–116,119–121,135,211)	Cohort	7	HIC	31	AQ	31
			Case–control	29	LMIC	5	IQ	5
>12 months vs. Never								
50	50	(38–40,42,48,51,56,57,59–67,69,71,74,78–80,82,83,85,88,93,95,96,98,99,101,105,107,110,112–116,119–121,125–127,131,135,211)	Cohort	8	HIC	43	AQ	41
			Case–control	42	LMIC	7	IQ	9

HIC, high-income country; LIC, low-income country; AQ, adequate quality; IQ, inadequate quality.

**Table A3:** Summary of studies included in ovarian carcinoma

Estimates	Studies	Ref. No.	Design		Country		Quality	
Ever vs. Never								
41	40	(65,69,136–173)	Cohort	5	HIC	35	AQ	25
			Case–control	35	LMIC	5	IQ	15
<6 months vs. Never								
20	19	(137,138,142,144,147–150,153,155,158,161,163,165–168,170,173)	Cohort	3	HIC	18	AQ	16
			Case–control	16	LMIC	1	IQ	3
6–12 months vs. Never								
19	18	(137,138,142,144,147,148,150,153,155,158,161,163,165–168,170,173)	Cohort	2	HIC	17	AQ	15
			Case–control	16	LMIC	1	IQ	3
>12 months vs. Never								
29	28	(65,69,137–140,142–144,146–148,150,152,153,155,158,161,163–168,170–173)	Cohort	3	HIC	24	AQ	23
			Case–control	25	LMIC	4	IQ	5

LIC, low-income country; AQ, adequate quality; IQ, inadequate quality.

## Abbreviations

CI: Confidence interval
HIC: High-income country
LMIC: Low- and middle-income country
MeSH: Medical Subject Heading
OR: Odds ratio
PPD: Postpartum depression
RCTs: Randomised controlled trials
RR: Relative risk
SMD: Standardised mean difference
UNICEF: United Nations Children's Fund
WHO: World Health Organization

## References

[1] WHO (2009). Infant and Young Child feeding. Model Chapter for textbooks for medical students and allied health professionals.

[2] Brion MJ, Lawlor DA, Matijasevich A, Horta B, Anselmi L, Araujo CL (2011). What are the causal effects of breastfeeding on IQ, obesity and blood pressure? Evidence from comparing high-income with middle-income cohorts. Int J Epidemiol.

[3] Gonzalez-Jimenez E, Garcia PA, Aguilar MJ, Padilla CA, Alvarez J (2014). Breastfeeding and the prevention of breast cancer: a retrospective review of clinical histories. J Clin Nurs.

[4] Ip S, Chung M, Raman G, Trikalinos TA, Lau J (2009). A summary of the Agency for Healthcare Research and Quality's evidence report on breastfeeding in developed countries. Breastfeed Med.

[5] Haiek LN, Kramer MS, Ciampi A, Tirado R (2001). Postpartum weight loss and infant feeding. J Am Board Fam Pract.

[6] Ebina S, Kashiwakura I (2012). Influence of breastfeeding on maternal blood pressure at one month postpartum *Int*. J Womens Health.

[7] Lipworth L, Bailey LR, Trichopoulos D (2000). History of breast-feeding in relation to breast cancer risk: a review of the epidemiologic literature. J Natl Cancer Inst.

[8] Britt K, Ashworth A, Smalley M (2007). Pregnancy and the risk of breast cancer. Endocr Relat Cancer.

[9] Siegel R, Naishadham D, Jemal A (2012). Cancer statistics, 2012. CA Cancer J Clin.

[10] Bray F, Loos AH, Tognazzo S, La Vecchia C (2005). Ovarian cancer in Europe: cross-sectional trends in incidence and mortality in 28 countries, 1953-2000. Int J Cancer.

[11] Sueblinvong T, Carney ME (2009). Current understanding of risk factors for ovarian cancer. Curr Treat Options Oncol.

[12] Whittemore AS, Harris R, Itnyre J (1992). Characteristics relating to ovarian cancer risk: collaborative analysis of 12 US case-control studies. IV. The pathogenesis of epithelial ovarian cancer. Collaborative Ovarian Cancer Group. Am J Epidemiol.

[13] Kovacs CS, Kronenberg HM (1997). Maternal-fetal calcium and bone metabolism during pregnancy, puerperium, and lactation. Endocr Rev.

[14] Stuebe AM, Rich-Edwards JW, Willett WC, Manson JE, Michels KB (2005). Duration of lactation and incidence of type 2 diabetes. JAMA.

[15] Schwarz EB, Brown JS, Creasman JM, Stuebe A, McClure CK, Van Den Eeden SK (2010). Lactation and maternal risk of type 2 diabetes: a population-based study. Am J Med.

[16] Gray RH, Campbell OM, Apelo R, Eslami SS, Zacur H, Ramos RM (1990). Risk of ovulation during lactation. Lancet.

[17] Andersen AN, Schioler V (1982). Influence of breast-feeding pattern on pituitary-ovarian axis of women in an industrialized community. Am J Obstet Gynecol.

[18] Rivera R, Kennedy KI, Ortiz E, Barrera M, Bhiwandiwala PP (1988). Breast-feeding and the return to ovulation in Durango, Mexico. Fertil Steril.

[19] Diaz S, Rodriguez G, Peralta O, Miranda P, Casado ME, Salvatierra AM (1988). Lactational amenorrhea and the recovery of ovulation and fertility in fully nursing Chilean women. Contraception.

[20] Halbreich U, Karkun S (2006). Cross-cultural and social diversity of prevalence of postpartum depression and depressive symptoms. J Affect Disord.

[21] Josefsson A, Sydsjo G (2007). A follow-up study of postpartum depressed women: recurrent maternal depressive symptoms and child behavior after four years. Arch Womens Ment Health.

[22] Pincus HA, Pettit AR (2001). The societal costs of chronic major depression. J Clin Psychiatry.

[23] Ystrom E (2012). Breastfeeding cessation and symptoms of anxiety and depression: a longitudinal cohort study. BMC Pregnancy Childbirth.

[24] Chung EK, McCollum KF, Elo IT, Lee HJ, Culhane JF (2004). Maternal depressive symptoms and infant health practices among low-income women. Pediatrics.

[25] Linne Y, Barkeling B, Rossner S (2002). Long-term weight development after pregnancy. Obes Rev.

[26] Rooney BL, Schauberger CW, Mathiason MA (2005). Impact of perinatal weight change on long-term obesity and obesity-related illnesses. Obstet Gynecol.

[27] Stuebe AM, Rich-Edwards JW (2009). The reset hypothesis: lactation and maternal metabolism. Am J Perinatol.

[28] Excellence NIoHaC Donor milk banks: the operation of donor milk bank services. NICE 2010.

[29] Lefebvre C, Manheimer E, Glanville J, Higgins JPT, Greene S (2008). Searching for studies. Cochrane Handbook for Systematic Reviews of Interventions, Version 5.0.

[30] Luan NN, Wu QJ, Gong TT, Vogtmann E, Wang YL, Lin B (2013). Breastfeeding and ovarian cancer risk: a meta-analysis of epidemiologic studies. Am J Clin Nutr.

[31] Aune D, Norat T, Romundstad P, Vatten LJ (2014). Breastfeeding and the maternal risk of type 2 diabetes: a systematic review and dose-response meta-analysis of cohort studies. Nutr Metab Cardiovasc Dis.

[32] Dias CC, Figueiredo B (2015). Breastfeeding and depression: a systematic review of the literature. J Affect Disord.

[33] Neville CE, McKinley MC, Holmes VA, Spence D, Woodside JV (2014). The relationship between breastfeeding and postpartum weight change–a systematic review and critical evaluation. Int J Obes (Lond).

[34] Harris R, Bradburn M, Deeks J, Harbord R, Altman D, Sterne J (2008). metan: fixed- and random-effects meta-analysis. Stata J.

[35] World Bank (2014). Low and middle income country data.

[36] Sterne JAC, Higgins JPT, Reeves BC on behalf of the Development Group for ACROBAT-NRSI

[37] Sterne JAC, Egger M, Smith GD (2001). Investigating and Dealing with Publication and Other Biases, in Systematic Reviews in Health Care: Meta-Analysis in Context.

[38] United Kingdom National Case-Control Study Group (1993). Breast feeding and risk of breast cancer in young women. United Kingdom National Case-Control Study Group. BMJ.

[39] Adami HO, Bergstrom R, Lund E, Meirik O (1990). Absence of association between reproductive variables and the risk of breast cancer in young women in Sweden and Norway. Br J Cancer.

[40] Akbari A, Razzaghi Z, Homaee F, Khayamzadeh M, Movahedi M, Akbari ME (2011). Parity and breastfeeding are preventive measures against breast cancer in Iranian women. Breast Cancer.

[41] Ambrosone CB, Zirpoli G, Ruszczyk M, Shankar J, Hong CC, McIlwain D (2014). Parity and breastfeeding among African-American women: differential effects on breast cancer risk by estrogen receptor status in the Women's Circle of Health Study. Cancer Causes Control.

[42] Andrieu N, Goldgar DE, Easton DF, Rookus M, Brohet R, Antoniou AC (2006). Pregnancies, breast-feeding, and breast cancer risk in the International BRCA1/2 Carrier Cohort Study (IBCCS). J Natl Cancer Inst.

[43] Awatef M, Olfa G, Imed H, Kacem M, Imen C, Rim C (2010). Breastfeeding reduces breast cancer risk: a case-control study in Tunisia. Cancer Causes Control.

[44] Bao PP, Shu XO, Gao YT, Zheng Y, Cai H, Deming SL (2011). Association of hormone-related characteristics and breast cancer risk by estrogen receptor/progesterone receptor status in the shanghai breast cancer study. Am J Epidemiol.

[45] Barba M, McCann SE, Nie J, Vito D, Stranges S, Fuhrman B (2006). Perinatal exposures and breast cancer risk in the Western New York Exposures and Breast Cancer (WEB) Study. Cancer Causes Control.

[46] Brignone G, Cusimano R, Dardanoni G, Gugliuzza M, Lanzarone F, Scibilia V (1987). A case-control study on breast cancer risk factors in a southern European population. Int J Epidemiol.

[47] Brinton LA, Hoover R, Fraumeni JF (1983). Reproductive factors in the aetiology of breast cancer. Br J Cancer.

[48] Brinton LA, Potischman NA, Swanson CA, Schoenberg JB, Coates RJ, Gammon MD (1995). Breastfeeding and breast cancer risk. Cancer Causes Control.

[49] Butt Z, Haider SF, Arif S, Khan MR, Ashfaq U, Shahbaz U (2012). Breast cancer risk factors: a comparison between pre-menopausal and post-menopausal women. J Pak Med Assoc.

[50] Calderon-Garciduenas AL, Paras-Barrientos FU, Cardenas-Ibarra L, Gonzalez-Guerrero JF, Villarreal-Rios E, Staines-Boone T (2000). Risk factors of breast cancer in Mexican women. Salud Publica Mex.

[51] Chang-Claude J, Eby N, Kiechle M, Bastert G, Becher H (2000). Breastfeeding and breast cancer risk by age 50 among women in Germany. Cancer Causes Control.

[52] Chung S, Park SK, Sung H, Song N, Han W, Noh DY (2013). Association between chronological change of reproductive factors and breast cancer risk defined by hormone receptor status: results from the Seoul Breast Cancer Study. Breast Cancer Res Treat.

[53] Coogan PF, Rosenberg L, Shapiro S, Hoffman M (1999). Lactation and breast carcinoma risk in a South African population. Cancer.

[54] De Silva M, Senarath U, Gunatilake M, Lokuhetty D (2010). Prolonged breastfeeding reduces risk of breast cancer in Sri Lankan women: a case-control study. Cancer Epidemiol.

[55] Duffy SW, Roberts MM, Elton RA (1983). Risk factors for breast cancer: relevance to screening. J Epidemiol Community Health.

[56] Enger SM, Ross RK, Henderson B, Bernstein L (1997). Breastfeeding history, pregnancy experience and risk of breast cancer. Br J Cancer.

[57] Enger SM, Ross RK, Paganini-Hill A, Bernstein L (1998). Breastfeeding experience and breast cancer risk among postmenopausal women. Cancer Epidemiol Biomarkers Prev.

[58] Faheem M, Khurram M, Jafri IA, Mehmood H, Hasan Z, Iqbal GS (2007). Risk factors for breast cancer in patients treated at NORI Hospital, Islamabad. J Pak Med Assoc.

[59] Freudenheim JL, Marshall JR, Vena JE, Moysich KB, Muti P, Laughlin R (1997). Lactation history and breast cancer risk. Am J Epidemiol.

[60] Furberg H, Newman B, Moorman P, Millikan R (1999). Lactation and breast cancer risk. Int J Epidemiol.

[61] Gajalakshmi V, Mathew A, Brennan P, Rajan B, Kanimozhi VC, Mathews A (2009). Breastfeeding and breast cancer risk in India: a multicenter case-control study. Int J Cancer.

[62] Gammon MD, Neugut AI, Santella RM, Teitelbaum SL, Britton JA, Terry MB (2002). The Long Island Breast Cancer Study Project: description of a multi-institutional collaboration to identify environmental risk factors for breast cancer. Breast Cancer Res Treat.

[63] Gao YT, Shu XO, Dai Q, Potter JD, Brinton LA, Wen W (2000). Association of menstrual and reproductive factors with breast cancer risk: results from the Shanghai Breast Cancer Study. Int J Cancer.

[64] Gilliland FD, Hunt WC, Baumgartner KB, Crumley D, Nicholson CS, Fetherolf J (1998). Reproductive risk factors for breast cancer in Hispanic and non-Hispanic white women: the New Mexico Women's Health Study. Am J Epidemiol.

[65] Gronwald J, Byrski T, Huzarski T, Cybulski C, Sun P, Tulman A (2006). Influence of selected lifestyle factors on breast and ovarian cancer risk in BRCA1 mutation carriers from Poland. Breast Cancer Res Treat.

[66] Hadjisavvas A, Loizidou MA, Middleton N, Michael T, Papachristoforou R, Kakouri E (2010). An investigation of breast cancer risk factors in Cyprus: a case control study. BMC Cancer.

[67] Hajian-Tilaki KO, Kaveh-Ahangar T (2011). Reproductive factors associated with breast cancer risk in northern Iran. Med Oncol.

[68] Hejar AR, Chong FB, Rosnan H, Zailina H (2004). Breast cancer and lifestyle risks among Chinese women in the Klang Valley in 2001. Med J Malaysia.

[69] Hirose K, Tajima K, Hamajima N, Kuroishi T, Kuzuya K, Miura S (1999). Comparative case-referent study of risk factors among hormone-related female cancers in Japan. Jpn J Cancer Res.

[70] Huang WY, Newman B, Millikan RC, Conway K, Hulka BS, Schell MJ (2000). Risk of breast cancer according to the status of HER-2/neu oncogene amplification. Cancer Epidemiol Biomarkers Prev.

[71] Inumaru LE, Irineu Gomes Duarte Quintanilha M, Aparecida da Silveira E, Veloso Naves MM (2012). Risk and protective factors for breast cancer in Midwest of Brazil. J Environ Public Health.

[72] Iwasaki M, Otani T, Inoue M, Sasazuki S, Tsugane S (2007). Role and impact of menstrual and reproductive factors on breast cancer risk in Japan. Eur J Cancer Prev.

[73] Kamarudin R, Shah SA, Hidayah N (2006). Lifestyle factors and breast cancer: a case-control study in Kuala Lumpur, Malaysia. Asian Pac J Cancer Prev.

[74] Katsouyanni K, Lipworth L, Trichopoulou A, Samoli E, Stuver S, Trichopoulos D (1996). A case-control study of lactation and cancer of the breast. Br J Cancer.

[75] Kawai M, Minami Y, Kuriyama S, Kakizaki M, Kakugawa Y, Nishino Y (2010). Reproductive factors, exogenous female hormone use and breast cancer risk in Japanese: the Miyagi Cohort Study. Cancer Causes Control.

[76] Kim Y, Choi JY, Lee KM, Park SK, Ahn SH, Noh DY (2007). Dose-dependent protective effect of breast-feeding against breast cancer among ever-lactated women in Korea. Eur J Cancer Prev.

[77] Kishk NA (1999). Breast cancer in relation to some reproductive factors. J Egypt Public Health Assoc.

[78] Kruk J (2007). Association of lifestyle and other risk factors with breast cancer according to menopausal status: a case-control study in the Region of Western Pomerania (Poland). Asian Pac J Cancer Prev.

[79] Kuru B, Ozaslan C, Ozdemir P, Dinc S, Camlibel M, Alagol H (2002). Risk factors for breast cancer in Turkish women with early pregnancies and long-lasting lactation–a case-control study. Acta Oncol.

[80] Layde PM, Webster LA, Baughman AL, Wingo PA, Rubin GL, Ory HW (1989). The independent associations of parity, age at first full term pregnancy, and duration of breastfeeding with the risk of breast cancer. Cancer and Steroid Hormone Study Group. J Clin Epidemiol.

[81] Lee HP, Gourley L, Duffy SW, Esteve J, Lee J, Day NE (1992). Risk factors for breast cancer by age and menopausal status: a case-control study in Singapore. Cancer Causes Control.

[82] Lee SY, Kim MT, Kim SW, Song MS, Yoon SJ (2003). Effect of lifetime lactation on breast cancer risk: a Korean women's cohort study. Int J Cancer.

[83] Li CI, Beaber EF, Tang MT, Porter PL, Daling JR, Malone KE (2013). Reproductive factors and risk of estrogen receptor positive, triple-negative, and HER2-neu overexpressing breast cancer among women 20-44 years of age. Breast Cancer Res Treat.

[84] Liu YT, Gao CM, Ding JH, Li SP, Cao HX, Wu JZ (2011). Physiological, reproductive factors and breast cancer risk in Jiangsu province of China. Asian Pac J Cancer Prev.

[85] London SJ, Colditz GA, Stampfer MJ, Willett WC, Rosner BA, Corsano K (1990). Lactation and risk of breast cancer in a cohort of US women. Am J Epidemiol.

[86] Lord SJ, Bernstein L, Johnson KA, Malone KE, McDonald JA, Marchbanks PA (2008). Breast cancer risk and hormone receptor status in older women by parity, age of first birth, and breastfeeding: a case-control study. Cancer Epidemiol Biomarkers Prev.

[87] Lumachi F, Ermani M, Brandes AA, Basso U, Paris M, Basso SM (2002). Breast cancer risk in healthy and symptomatic women: results of a multivariate analysis. A case-control study. Biomed Pharmacother.

[88] MacMahon B, Purde M, Cramer D, Hint E (1982). Association of breast cancer risk with age at first and subsequent births: a study in the population of the Estonian Republic. J Natl Cancer Inst.

[89] Mahouri K, Dehghani Zahedani M, Zare S (2007). Breast cancer risk factors in south of Islamic Republic of Iran: a case-control study. East Mediterr Health J.

[90] Marcus PM, Baird DD, Millikan RC, Moorman PG, Qaqish B, Newman B (1999). Adolescent reproductive events and subsequent breast cancer risk. Am J Public Health.

[91] Matalqah L, Radaideh K, Yusoff ZM, Awaisu A (2011). Predictors of breast cancer among women in a northern state of Malaysia: a matched case-control study. Asian Pac J Cancer Prev.

[92] McKenzie F, Ellison-Loschmann L, Jeffreys M, Firestone R, Pearce N, Romieu I (2014). Healthy lifestyle and risk of breast cancer for indigenous and non-indigenous women in New Zealand: a case control study. BMC Cancer.

[93] McTiernan A, Thomas DB, Johnson LK, Roseman D (1986). Risk factors for estrogen receptor-rich and estrogen receptor-poor breast cancers. J Natl Cancer Inst.

[94] Meeske K, Press M, Patel A, Bernstein L (2004). Impact of reproductive factors and lactation on breast carcinoma in situ risk. Int J Cancer.

[95] Michels KB, Willett WC, Rosner BA, Manson JE, Hunter DJ, Colditz GA (1996). Prospective assessment of breastfeeding and breast cancer incidence among 89,887 women. Lancet.

[96] Minami Y, Ohuchi N, Fukao A, Hisamichi S (1997). Risk factors for breast cancer: a case-control study of screen-detected breast cancer in Miyagi Prefecture, Japan. Breast Cancer Res Treat.

[97] Morales L, Alvarez-Garriga C, Matta J, Ortiz C, Vergne Y, Vargas W (2013). Factors associated with breast cancer in Puerto Rican women. J Epidemiol Glob Health.

[98] Negri E, Braga C, La Vecchia C, Levi F, Talamini R, Franceschi S (1996). Lactation and the risk of breast cancer in an Italian population. Int J Cancer.

[99] Newcomb PA, Storer BE, Longnecker MP, Mittendorf R, Greenberg ER, Clapp RW (1994). Lactation and a reduced risk of premenopausal breast cancer. N Engl J Med.

[100] Olaya-Contreras P, Pierre B, Lazcano-Ponce E, Villamil-Rodriguez J, Posso-Valencia HJ (1999). [Reproductive risk factors associated with breast cancer in Colombian women]. Rev Saude Publica.

[101] Oran B, Celik I, Erman M, Baltali E, Zengin N, Demirkazik F (2004). Analysis of menstrual, reproductive, and life-style factors for breast cancer risk in Turkish women: a case-control study. Med Oncol.

[102] Ortiz Mendoza CM, Galvan Martinez EA (2007). [Reproductive risk factors of breast cancer in patients attended at a second level urban hospital]. Ginecol Obstet Mex.

[103] Ozmen V, Ozcinar B, Karanlik H, Cabioglu N, Tukenmez M, Disci R (2009). Breast cancer risk factors in Turkish women–a University Hospital based nested case control study. World J Surg Oncol.

[104] Palmer JR, Viscidi E, Troester MA, Hong CC, Schedin P, Bethea TN (2014). Parity, lactation, and breast cancer subtypes in African American women: results from the AMBER Consortium. J Natl Cancer Inst.

[105] Paul C, Skegg DC, Spears GF (1990). Oral contraceptives and risk of breast cancer. Int J Cancer.

[106] Peterson NB, Huang Y, Newcomb PA, Titus-Ernstoff L, Trentham-Dietz A, Anic G (2008). Childbearing recency and modifiers of premenopausal breast cancer risk. Cancer Epidemiol Biomarkers Prev.

[107] Phipps AI, Chlebowski RT, Prentice R, McTiernan A, Wactawski-Wende J, Kuller LH (2011). Reproductive history and oral contraceptive use in relation to risk of triple-negative breast cancer. J Natl Cancer Inst.

[108] Purwanto H, Sadjimin T, Dwiprahasto I (2000). Lactation and the risk of breast cancer. Gan To Kagaku Ryoho.

[109] Rao DN, Ganesh B, Desai PB (1994). Role of reproductive factors in breast cancer in a low-risk area: a case-control study. Br J Cancer.

[110] Romieu I, Hernandez-Avila M, Lazcano E, Lopez L, Romero-Jaime R (1996). Breast cancer and lactation history in Mexican women. Am J Epidemiol.

[111] Rookus MA, van Leeuwen FE (1994). Oral contraceptives and risk of breast cancer in women aged 20-54 years. Netherlands Oral Contraceptives and Breast Cancer Study Group. Lancet.

[112] Shantakumar S, Terry MB, Teitelbaum SL, Britton JA, Millikan RC, Moorman PG (2007). Reproductive factors and breast cancer risk among older women. Breast Cancer Res Treat.

[113] Shema L, Ore L, Ben-Shachar M, Haj M, Linn S (2007). The association between breastfeeding and breast cancer occurrence among Israeli Jewish women: a case control study. J Cancer Res Clin Oncol.

[114] Siskind V, Schofield F, Rice D, Bain C (1989). Breast cancer and breastfeeding: results from an Australian case-control study. Am J Epidemiol.

[115] Stuebe AM, Willett WC, Xue F, Michels KB (2009). Lactation and incidence of premenopausal breast cancer: a longitudinal study. Arch Intern Med.

[116] Stuver SO, Hsieh CC, Bertone E, Trichopoulos D (1997). The association between lactation and breast cancer in an international case-control study: a re-analysis by menopausal status. Int J Cancer.

[117] Sugawara Y, Kakizaki M, Nagai M, Tomata Y, Hoshi R, Watanabe I (2013). Lactation pattern and the risk for hormone-related female cancer in Japan: the Ohsaki Cohort Study. Eur J Cancer Prev.

[118] Tao SC, Yu MC, Ross RK, Xiu KW (1988). Risk factors for breast cancer in Chinese women of Beijing. Int J Cancer.

[119] Tessaro S, Beria JU, Tomasi E, Victora CG (2003). Breastfeeding and breast cancer: a case-control study in Southern Brazil. Cad Saude Publica.

[120] Thomas DB, Noonan EA (1993). Breast cancer and prolonged lactation. The WHO Collaborative Study of Neoplasia and Steroid Contraceptives. Int J Epidemiol.

[121] Tryggvadottir L, Tulinius H, Eyfjord JE, Sigurvinsson T (2001). Breastfeeding and reduced risk of breast cancer in an Icelandic cohort study. Am J Epidemiol.

[122] Ursin G, Bernstein L, Wang Y, Lord SJ, Deapen D, Liff JM (2004). Reproductive factors and risk of breast carcinoma in a study of white and African-American women. Cancer.

[123] Wakai K, Ohno Y, Watanabe S, Sakamoto G, Kasumi F, Suzuki S (1994). Risk factors for breast cancer among Japanese women in Tokyo: a case-control study. J Epidemiol.

[124] Wang QS, Ross RK, Yu MC, Ning JP, Henderson BE, Kimm HT (1992). A case-control study of breast cancer in Tianjin, China. Cancer Epidemiol Biomarkers Prev.

[125] Warner ET, Colditz GA, Palmer JR, Partridge AH, Rosner BA, Tamimi RM (2013). Reproductive factors and risk of premenopausal breast cancer by age at diagnosis: are there differences before and after age 40?. Breast Cancer Res Treat.

[126] White E, Malone KE, Weiss NS, Daling JR (1994). Breast cancer among young U.S. women in relation to oral contraceptive use. J Natl Cancer Inst.

[127] Work ME, John EM, Andrulis IL, Knight JA, Liao Y, Mulligan AM (2014). Reproductive risk factors and oestrogen/progesterone receptor-negative breast cancer in the Breast Cancer Family Registry. Br J Cancer.

[128] Wu AH, Ziegler RG, Pike MC, Nomura AM, West DW, Kolonel LN (1996). Menstrual and reproductive factors and risk of breast cancer in Asian-Americans. Br J Cancer.

[129] Xing P, Li J, Jin F (2010). A case-control study of reproductive factors associated with subtypes of breast cancer in Northeast China. Med Oncol.

[130] Yang CP, Weiss NS, Band PR, Gallagher RP, White E, Daling JR (1993). History of lactation and breast cancer risk. Am J Epidemiol.

[131] Yavari P, Mosavizadeh M, Sadrol-Hefazi B, Mehrabi Y (2005). Reproductive characteristics and the risk of breast cancer–a case-control study in Iran. Asian Pac J Cancer Prev.

[132] Yoo KY, Tajima K, Kuroishi T, Hirose K, Yoshida M, Miura S (1992). Independent protective effect of lactation against breast cancer: a case-control study in Japan. Am J Epidemiol.

[133] Yuan JM, Yu MC, Ross RK, Gao YT, Henderson BE (1988). Risk factors for breast cancer in Chinese women in Shanghai. Cancer Res.

[134] Zheng T, Duan L, Liu Y, Zhang B, Wang Y, Chen Y (2000). Lactation reduces breast cancer risk in Shandong Province, China. Am J Epidemiol.

[135] Zheng T, Holford TR, Mayne ST, Owens PH, Zhang Y, Zhang B (2001). Lactation and breast cancer risk: a case-control study in Connecticut. Br J Cancer.

[136] The Cancer and Steroid Hormone Study of the Centers for Disease Control and the National Institute of Child Health and Human Development (1987). The reduction in risk of ovarian cancer associated with oral-contraceptive use. N Engl J Med.

[137] Antoniou AC, Rookus M, Andrieu N, Brohet R, Chang-Claude J, Peock S (2009). Reproductive and hormonal factors, and ovarian cancer risk for BRCA1 and BRCA2 mutation carriers: results from the International BRCA1/2 Carrier Cohort Study. Cancer Epidemiol Biomarkers Prev.

[138] Booth M, Beral V, Smith P (1989). Risk factors for ovarian cancer: a case-control study. Br J Cancer.

[139] Chen Y, Wu PC, Lang JH, Ge WJ, Hartge P, Brinton LA (1992). Risk factors for epithelial ovarian cancer in Beijing, China. Int J Epidemiol.

[140] Chiaffarino F, Pelucchi C, Negri E, Parazzini F, Franceschi S, Talamini R (2005). Breastfeeding and the risk of epithelial ovarian cancer in an Italian population. Gynecol Oncol.

[141] Cramer DW, Hutchison GB, Welch WR, Scully RE, Ryan KJ (1983). Determinants of ovarian cancer risk. I. Reproductive experiences and family history. J Natl Cancer Inst.

[142] Danforth KN, Tworoger SS, Hecht JL, Rosner BA, Colditz GA, Hankinson SE (2007). Breastfeeding and risk of ovarian cancer in two prospective cohorts. Cancer Causes Control.

[143] Greggi S, Parazzini F, Paratore MP, Chatenoud L, Legge F, Mancuso S (2000). Risk factors for ovarian cancer in central Italy. Gynecol Oncol.

[144] Gwinn ML, Lee NC, Rhodes PH, Layde PM, Rubin GL (1990). Pregnancy, breast feeding, and oral contraceptives and the risk of epithelial ovarian cancer. J Clin Epidemiol.

[145] Harlow BL, Weiss NS, Roth GJ, Chu J, Daling JR (1988). Case-control study of borderline ovarian tumors: reproductive history and exposure to exogenous female hormones. Cancer Res.

[146] Hartge P, Schiffman MH, Hoover R, McGowan L, Lesher L, Norris HJ (1989). A case-control study of epithelial ovarian cancer. Am J Obstet Gynecol.

[147] Huusom LD, Frederiksen K, Hogdall EV, Glud E, Christensen L, Hogdall CK (2006). Association of reproductive factors, oral contraceptive use and selected lifestyle factors with the risk of ovarian borderline tumors: a Danish case-control study. Cancer Causes Control.

[148] Jordan SJ, Cushing-Haugen KL, Wicklund KG, Doherty JA, Rossing MA (2012). Breast-feeding and risk of epithelial ovarian cancer. Cancer Causes Control.

[149] Jordan SJ, Green AC, Whiteman DC, Webb PM (2007). Risk factors for benign, borderline and invasive mucinous ovarian tumors: epidemiological evidence of a neoplastic continuum?. Gynecol Oncol.

[150] Kurta ML, Moysich KB, Weissfeld JL, Youk AO, Bunker CH, Edwards RP (2012). Use of fertility drugs and risk of ovarian cancer: results from a U.S.-based case-control study. Cancer Epidemiol Biomarkers Prev.

[151] Le DC, Kubo T, Fujino Y, Sokal DC, Vach TH, Pham TM (2012). Reproductive factors in relation to ovarian cancer: a case-control study in Northern Vietnam. Contraception.

[152] McLaughlin JR, Risch HA, Lubinski J, Moller P, Ghadirian P, Lynch H (2007). Reproductive risk factors for ovarian cancer in carriers of BRCA1 or BRCA2 mutations: a case-control study. Lancet Oncol.

[153] Mills PK, Riordan DG, Cress RD (2004). Epithelial ovarian cancer risk by invasiveness and cell type in the Central Valley of California. Gynecol Oncol.

[154] Mink PJ, Folsom AR, Sellers TA, Kushi LH (1996). Physical activity, waist-to-hip ratio, and other risk factors for ovarian cancer: a follow-up study of older women. Epidemiology.

[155] Moorman PG, Calingaert B, Palmieri RT, Iversen ES, Bentley RC, Halabi S (2008). Hormonal risk factors for ovarian cancer in premenopausal and postmenopausal women. Am J Epidemiol.

[156] Mori M, Harabuchi I, Miyake H, Casagrande JT, Henderson BE, Ross RK (1988). Reproductive, genetic, and dietary risk factors for ovarian cancer. Am J Epidemiol.

[157] Mori M, Nishida T, Sugiyama T, Komai K, Yakushiji M, Fukuda K (1998). Anthropometric and other risk factors for ovarian cancer in a case-control study. Jpn J Cancer Res.

[158] Ness RB, Grisso JA, Klapper J, Schlesselman JJ, Silberzweig S, Vergona R (2000). Risk of ovarian cancer in relation to estrogen and progestin dose and use characteristics of oral contraceptives. SHARE Study Group. Steroid Hormones and Reproductions. Am J Epidemiol.

[159] Permuth-Wey J, Chen YA, Tsai YY, Chen Z, Qu X, Lancaster JM (2011). Inherited variants in mitochondrial biogenesis genes may influence epithelial ovarian cancer risk. Cancer Epidemiol Biomarkers Prev.

[160] Pieta B, Chmaj-Wierzchowska K, Opala T (2012). Past obstetric history and risk of ovarian cancer. Ann Agric Environ Med.

[161] Riman T, Dickman PW, Nilsson S, Correia N, Nordlinder H, Magnusson CM (2001). Risk factors for epithelial borderline ovarian tumors: results of a Swedish case-control study. Gynecol Oncol.

[162] Rosenblatt KA, Thomas DB (1993). Lactation and the risk of epithelial ovarian cancer. The WHO Collaborative Study of Neoplasia and Steroid Contraceptives. Int J Epidemiol.

[163] Rossing MA, Tang MT, Flagg EW, Weiss LK, Wicklund KG (2004). A case-control study of ovarian cancer in relation to infertility and the use of ovulation-inducing drugs. Am J Epidemiol.

[164] Salazar-Martinez E, Lazcano-Ponce EC, Gonzalez Lira-Lira G, Escudero-De los Rios P, Salmeron-Castro J, Hernandez-Avila M (1999). Reproductive factors of ovarian and endometrial cancer risk in a high fertility population in Mexico. Cancer Res.

[165] Siskind V, Green A, Bain C, Purdie D (1997). Breastfeeding, menopause, and epithelial ovarian cancer. Epidemiology.

[166] Titus-Ernstoff L, Rees JR, Terry KL, Cramer DW (2010). Breast-feeding the last born child and risk of ovarian cancer. Cancer Causes Control.

[167] Tsilidis KK, Allen NE, Key TJ, Dossus L, Lukanova A, Bakken K (2011). Oral contraceptive use and reproductive factors and risk of ovarian cancer in the European Prospective Investigation into Cancer and Nutrition. Br J Cancer.

[168] Tung KH, Goodman MT, Wu AH, McDuffie K, Wilkens LR, Kolonel LN (2003). Reproductive factors and epithelial ovarian cancer risk by histologic type: a multiethnic case-control study. Am J Epidemiol.

[169] Weiderpass E, Sandin S, Inoue M, Shimazu T, Iwasaki M, Sasazuki S (2012). Risk factors for epithelial ovarian cancer in Japan – results from the Japan Public Health Center-based Prospective Study cohort. Int J Oncol.

[170] Whittemore AS, Harris R, Itnyre J (1992). Characteristics relating to ovarian cancer risk: collaborative analysis of 12 US case-control studies. II. Invasive epithelial ovarian cancers in white women. Collaborative Ovarian Cancer Group. Am J Epidemiol.

[171] Wilailak S, Vipupinyo C, Suraseranivong V, Chotivanich K, Kietpeerakool C, Tanapat Y (2012). Depot medroxyprogesterone acetate and epithelial ovarian cancer: a multicentre case-control study. BJOG.

[172] Yen ML, Yen BL, Bai CH, Lin RS (2003). Risk factors for ovarian cancer in Taiwan: a case-control study in a low-incidence population. Gynecol Oncol.

[173] Zhang M, Xie X, Lee AH, Binns CW (2004). Prolonged lactation reduces ovarian cancer risk in Chinese women. Eur J Cancer Prev.

[174] Chowdhury S, Sarkar NR, Roy SK (2002). Impact of lactational performance on bone mineral density in marginally-nourished Bangladeshi women. J Health Popul Nutr.

[175] Drinkwater BL, Chesnut CH (1991). Bone density changes during pregnancy and lactation in active women: a longitudinal study. Bone Miner.

[176] Hawker GA, Forsmo S, Cadarette SM, Schei B, Jaglal SB, Forsen L (2002). Correlates of forearm bone mineral density in young Norwegian women: the Nord-Trondelag Health Study. Am J Epidemiol.

[177] Henderson PH, Sowers M, Kutzko KE, Jannausch ML (2000). Bone mineral density in grand multiparous women with extended lactation. Am J Obstet Gynecol.

[178] Lenora J, Lekamwasam S, Karlsson MK (2009). Effects of multiparity and prolonged breast-feeding on maternal bone mineral density: a community-based cross-sectional study. BMC Womens Health.

[179] Wiklund PK, Xu L, Wang Q, Mikkola T, Lyytikainen A, Volgyi E (2012). Lactation is associated with greater maternal bone size and bone strength later in life. Osteoporos Int.

[180] Benitez I, de la Cruz J, Suplido A, Oblepias V, Kennedy K, Visness C (1992). Extending lactational amenorrhoea in Manila: a successful breast-feeding education programme. J Biosoc Sci.

[181] Dada OA, Akesode FA, Olanrewaju DM, Olowu OA, Sule-Odu O, Fakoya TA (2002). Infant feeding and lactational amenorrhea in Sagamu, Nigeria. Afr J Reprod Health.

[182] Davies-Adetugbo AA, Ojofeitimi EO (1996). Maternal education, breastfeeding behaviours and lactational amenorrhoea: studies among two ethnic communities in Ile Ife, Nigeria. Nutr Health.

[183] Dewey KG, Cohen RJ, Rivera LL, Canahuati J, Brown KH (1997). Effects of age at introduction of complementary foods to breast-fed infants on duration of lactational amenorrhea in Honduran women. Am J Clin Nutr.

[184] Egbuonu I, Ezechukwu CC, Chukwuka JO, Ikechebelu JI (2005). Breast-feeding, return of menses, sexual activity and contraceptive practices among mothers in the first six months of lactation in Onitsha, South Eastern Nigeria. J Obstet Gynaecol.

[185] Ingram J, Hunt L, Woolridge M, Greenwood R (2004). The association of progesterone, infant formula use and pacifier use with the return of menstruation in breastfeeding women: a prospective cohort study. Eur J Obstet Gynecol Reprod Biol.

[186] Kumar S, Reddaiah VP (1988). Lactational amenorrhea in urban poor women and its implications for use of contraception. Indian Pediatr.

[187] Lewis PR, Brown JB, Renfree MB, Short RV (1991). The resumption of ovulation and menstruation in a well-nourished population of women breastfeeding for an extended period of time. Fertil Steril.

[188] Radwan H, Mussaiger AO, Hachem F (2009). Breast-feeding and lactational amenorrhea in the United Arab Emirates. J Pediatr Nurs.

[189] Ravera M, Ravera C, Reggiori A, Cocozza E, Cianta F, Riccioni G (1995). A study of breastfeeding and the return of menses in Hoima District, Uganda. East Afr Med J.

[190] WHO (1998). The World Health Organization Multinational Study of Breast-feeding and Lactational Amenorrhea. I. Description of infant feeding patterns and of the return of menses. World Health Organization Task Force on Methods for the Natural Regulation of Fertility. Fertil Steril.

[191] Sarkar NR, Taylor R (2005). Weight loss during prolonged lactation in rural Bangladeshi mothers. J Health Popul Nutr.

[192] Stuebe AM, Kleinman K, Gillman MW, Rifas-Shiman SL, Gunderson EP, Rich-Edwards J (2010). Duration of lactation and maternal metabolism at 3 years postpartum. J Womens Health (Larchmt).

[193] Onyango AW, Nommsen-Rivers L, Siyam A, Borghi E, de Onis M, Garza C (2011). Post-partum weight change patterns in the WHO Multicentre Growth Reference Study. Matern Child Nutr.

[194] Monteiro da Silva Mda C, Marlucia Oliveira A, Pereira Magalhaes de Oliveira L, Silva dos Santos Fonseca DN, Portela de Santana ML, de Araujo Goes Neto E (2013). Determinants of postpartum weight variation in a cohort of adult women; a hierarchical approach. Nutr Hosp.

[195] Samano R, Martinez-Rojano H, Godinez Martinez E, Sanchez Jimenez B, Villeda Rodriguez GP, Perez Zamora J (2013). Effects of breastfeeding on weight loss and recovery of pregestational weight in adolescent and adult mothers. Food Nutr Bull.

[196] Sakai T (2001). Does breastfeeding reduce risk for breast cancer? A short lesson in evidence-based practice MCN. Am J Matern Child Nurs.

[197] Velie EM, Nechuta S, Osuch JR (2005). Lifetime reproductive and anthropometric risk factors for breast cancer in postmenopausal women. Breast Dis.

[198] Althuis MD, Fergenbaum JH, Garcia-Closas M, Brinton LA, Madigan MP, Sherman ME (2004). Etiology of hormone receptor-defined breast cancer: a systematic review of the literature. Cancer Epidemiol Biomarkers Prev.

[199] Ma H, Bernstein L, Pike MC, Ursin G (2006). Reproductive factors and breast cancer risk according to joint estrogen and progesterone receptor status: a meta-analysis of epidemiological studies. Breast Cancer Res.

[200] Newcomb PA (1997). Lactation and breast cancer risk. J Mammary Gland Biol Neoplasia.

[201] Collaborative Group on Hormonal Factors in Breast Cancer (2002). Breast cancer and breastfeeding: collaborative reanalysis of individual data from 47 epidemiological studies in 30 countries, including 50302 women with breast cancer and 96973 women without the disease. Lancet.

[202] Henderson BE, Feigelson HS (2000). Hormonal carcinogenesis. Carcinogenesis.

[203] Smalley M, Ashworth A (2003). Stem cells and breast cancer: a field in transit. Nat Rev Cancer.

[204] Jacob S, Spencer NA, Bullivant SB, Sellergren SA, Mennella JA, McClintock MK (2004). Effects of breastfeeding chemosignals on the human menstrual cycle. Hum Reprod.

[205] McNeilly AS (2001). Lactational control of reproduction. Reprod Fertil Dev.

[206] Miller SC, Bowman BM (2004). Rapid improvements in cortical bone dynamics and structure after lactation in established breeder rats. Anat Rec A Discov Mol Cell Evol Biol.

[207] Vekemans M (1997). Postpartum contraception: the lactational amenorrhea method. Eur J Contracept Reprod Health Care.

[208] Boardley DJ, Sargent RG, Coker AL, Hussey JR, Sharpe PA (1995). The relationship between diet, activity, and other factors, and postpartum weight change by race. Obstet Gynecol.

[209] Gunderson EP, Abrams B, Selvin S (2000). The relative importance of gestational gain and maternal characteristics associated with the risk of becoming overweight after pregnancy. Int J Obes Relat Metab Disord.

[210] WHO (1991). Indicators for assessing breastfeeding practices.

[211] Kabat GC, Kim MY, Woods NF, Habel LA, Messina CR, Wactawski-Wende J (2011). Reproductive and menstrual factors and risk of ductal carcinoma in situ of the breast in a cohort of postmenopausal women. Cancer Causes Control.

[212] Saeki T, Sano M, Komoike Y, Sonoo H, Honjyo H, Ochiai K (2008). No increase of breast cancer incidence in Japanese women who received hormone replacement therapy: overview of a case-control study of breast cancer risk in Japan. Int J Clin Oncol.

